# Fast MCMC sampling for hidden markov models to determine copy number variations

**DOI:** 10.1186/1471-2105-12-428

**Published:** 2011-11-02

**Authors:** Md Pavel Mahmud, Alexander Schliep

**Affiliations:** 1 Department of Computer Science, Rutgers University, 110 Frelinghuysen Road, Piscataway, NJ, 08854, USA; 2 BioMaPS Institute for Quantitative Biology, Rutgers University, 110 Frelinghuysen Road, Piscataway, NJ, 08854, USA

## Abstract

**Background:**

Hidden Markov Models (HMM) are often used for analyzing Comparative Genomic Hybridization (CGH) data to identify chromosomal aberrations or copy number variations by segmenting observation sequences. For efficiency reasons the parameters of a HMM are often estimated with maximum likelihood and a segmentation is obtained with the Viterbi algorithm. This introduces considerable uncertainty in the segmentation, which can be avoided with Bayesian approaches integrating out parameters using Markov Chain Monte Carlo (MCMC) sampling. While the advantages of Bayesian approaches have been clearly demonstrated, the likelihood based approaches are still preferred in practice for their lower running times; datasets coming from high-density arrays and next generation sequencing amplify these problems.

**Results:**

We propose an approximate sampling technique, inspired by compression of discrete sequences in HMM computations and by *kd*-trees to leverage spatial relations between data points in typical data sets, to speed up the MCMC sampling.

**Conclusions:**

We test our approximate sampling method on simulated and biological ArrayCGH datasets and high-density SNP arrays, and demonstrate a speed-up of 10 to 60 respectively 90 while achieving competitive results with the state-of-the art Bayesian approaches.

*Availability: *An implementation of our method will be made available as part of the open source GHMM library from http://ghmm.org.

## Background

The Sirens' call of Bayesian methods is that we can do without the parameters of models and, instead, compute probabilities of interest directly, indicating for example how likely a biological fact is given our data. The price one pays for that convenience is on one hand the conundrum of which prior distributions to choose and how to set their hyper-parameters; the frequent use of uniform priors is evidence that this is indeed non-trivial. On the other hand, unless the choice of likelihood and prior yields simple posteriors which we can integrate symbolically, we have to resort to sampling for example with Markov Chain Monte Carlo (MCMC) [1].

In the following we will concentrate on HMMs, stochastic functions of Markov Chains, which have not only been used extensively for discrete sequences--pairwise-sequence alignments with pair-HMMs [2], gene finding with labeled HMMs [3], and detecting remote homologs using profile HMMs [4]--but also for continuous-valued observations, such as gene expression time-courses [5]. Continuous observation sequences from either DNA microarrays or next generation sequencing experiments, note that the proportion of mapped reads in an interval is frequently used as a continuous measure of copy number, to detect chromosomal aberrations or copy number variations lead to the same fundamental computational problem and share characteristics of the data. The goal is to segment an observation sequence into regions in which there is little variation around a common mean. In other words, the assumption is that the data can be approximately described by piece-wise constant functions. Indeed, if hybridization intensity was an exact, un-biased measurement of DNA concentration before amplification, the sequence of hybridization intensities of probes along a chromosome would yield a piece-wise constant function in ArrayCGH experiments. This assumption holds true for a mixture of different cell populations because a finite sum of piece-wise constant functions is again a piece-wise constant function.

A wide range of methods for copy number detection in ArrayCGH data have been developed in recent years, including change-point detection based methods [6,7], smoothing based methods [8,9], and hierarchical clustering [10]. Here, we concentrate on HMM-based approaches which have been proposed for segmenting sequences of continuous-valued observations and shown to match or improve upon the state-of-the-art [11-13]. Once a model is trained from the data, either using maximum likelihood (ML) or maximum a posteriori (MAP), the segmentation is given by the most likely state sequence obtained with the Viterbi algorithm [14]. The segmentation, however, is highly dependent on the model parameters. A small change in the computed parameter values can adversely affect the recovered segmentation. A full Bayesian approach alleviates this dependence by integrating out the model parameters. As analytic integration of a complex high dimensional model is impossible for most distributions, the Bayesian approach requires the use of numerical integration techniques like MCMC [15], for example by direct Gibbs sampling [16] of model parameters and state paths. Scott [17] reports that combining the forward-backward recursions [18] and Gibbs sampling improves the converge rate and consequently the running time. Nevertheless, MCMC remains substantially slower than training one model and running Viterbi once and the loss in reliability introduced by relying on one ML or MAP model is ignored in practice. For discrete emissions, compressing sequences and computing forward and backward variables and Viterbi paths on the compressed sequences yields impressive speed-ups [19]. However, discretization of continuous emissions, similar to vector quantization used in speech recognition [18], is not viable as the separation between the different classes of observations is low since the observations are *one-dimensional*. Moreover, maximal compression is to be expected for small number of discrete symbols and clearly compression ratio conflicts with fidelity in the analysis.

**Figure 1:**
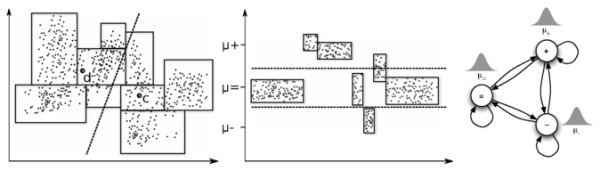
**Fundamental insight**. When reassigning two-dimensional data points to cluster centroids *c *and *d *in *k*-means clustering (left) the hyperrectangles obtained from *kd*-trees reduce the computational effort by making an argument about *all *points in an hyperrectangle based on their vertices; consider for example the rightmost hyperrectangle. For sequences (middle) there is no overlap in *y*-direction and decisions about the most likely state can be made per block considering the means of the Gaussians of a three-state HMM (right), *μ*_-_, *μ*_*= *_and *μ*_+_. Note that at every given block only a decision between the two states with closest mean is necessary, if one assume comparable variances. Decision boundaries are displayed dashed.

For a different task, arguments about spatial relations between groups of multi-variate data points were used to achieve considerable speed-up. Moore and colleagues used modified *kd*-trees, a data structure to efficiently execute spatial queries such as determining the nearest neighbor of a given point, to accelerate *k*-means [20]. The fundamental insight is illustrated in Figure 1 (left). In the reassignment step of *k*-means one has to find the nearest centroid for every data point. Due to the *kd*-tree, there are groups of points contained in a node of the tree for which this decision about the nearest centroid can be made *simultaneously *by a geometrical argument about the vertices of the hyperrectangle defined by this node. A similar *kd*-tree based approach was used in speech recognition [21,22] to quickly find the most important components in a mixture of large number of Gaussians and thus approximate the full observation density in one individual HMM state with multi-variate emissions.

At the core of our approach is a similar geometrical argument about several uni-variate data points based on a modified *kd*-tree. We adaptively identify blocks of observations, cf. Figure 1 (middle). For *all *observations in a block we now estimate, at least conceptually, the most likely state *simultaneously *depending on the means of the Gaussians in each state to gain a considerable speed-up proportional to the average block length. Similarly, we can avoid sampling states for each individual observation in a block if we can bound the posterior. Considerable care has to be taken for combining blocks and to bound the errors introduced by the approximations based on geometric arguments.

In summary, we

• propose the first use of spatial index structures for several consecutive observations and approximate computations based on geometric arguments to substantially speed-up the problem of segmenting sequences of continuous observations using HMM,

• demonstrate that very frequently used biological datasets of high relevance measuring chromosomal aberration and copy number variations are consistent with our assumptions of piece-wise constant observations with added noise, and

• achieve speed-ups between 10 and 90 on those biological datasets while maintaining competitive performance compared to the state-of-the-art.

## Methods

### HMM

We only consider HMMs with Gaussian emission distributions; see Figure 1 (right) for an example and [18] for an introduction. We will use the following notation: *N *denotes the number of states, *S = *{*s*_1_, *s*_2_, ..., *s*_*N*_} the set of states, *T *the length of an observation sequence *O *= {*o*_1_, *o*_2_, ..., *o*_*T*_} with *o*_*t *_∈ ℝ, *A = *{*a*_*ij*_}_1≤*i,j*≤*N *_the transition matrix, π = (π_1_, π_2_, ..., π_*N*_) the initial distribution over states, $B=\left\{\left(\mu_{1},\sigma_{1}^{2}\right),\ldots ,\left(\mu_{N},\sigma_{N}^{2}\right)\right\}$ with *μ*_1 _≤ ... ≤ *μ*_*N *_are parameters of the emission distributions, and *Q *= {*q*_1_, *q*_2_, ..., *q*_*T*_} the hidden state sequences with *q*_*t *_∈ *S*. From an observation sequence *O *we can obtain a maximum likelihood point estimate of the parameters (*A*, *B*, π) using the Expectation-Maximization (EM) or Baum-Welch [23] algorithm and compute the most likely path using the Viterbi algorithm [14].

### MCMC Sampling

Bayesian analysis requires choosing prior distributions on parameters and we use standard conjugate prior distributions following [1]. Specifically, we choose $\mu_{i}\sim N\left({\tilde{\mu }}_{i},{\tilde{\sigma }}_{i}\right),\sigma_{i}^{-2}\sim Gamma\left(a_{i},b_{i}\right),A_{i}\sim Dirichlet\left(\lambda^{A_{i}}\right)$, and π ~ *Dirichlet*(λ^π^), where ${\tilde{\mu }}_{i},\;{\tilde{\sigma }}_{i},\;a_{i},\;b_{i},\;\lambda^{A_{i}}$, and λ^π ^are the hyperparameters of the model.

It is only possible in some trivial cases to compute posterior distribution in closed form using analytic integration. In most applications, specially for high dimensional distributions, Monte Carlo integration techniques, like MCMC sampling by Gibbs sampling or Metropolis-Hastings, are easier to compute and generally produce more accurate results than numerical integration [15]. Scott [17] compares various MCMC approaches and strongly argues in favor of forward-backward Gibbs sampling (FBG-sampling), which has been successfully used by others [24,25], for it's excellent convergence characteristics. We briefly summarize FBG-sampling for an HMM λ = (*A*, *B*, π); see [17,26] for details:

1. Choose initial parameters *θ*^0 ^= (*A*^0^, *B*^0^, π^0^)

2. Perform the following steps for iteration 0 ≤ *m *<*M*.

(a) Compute *forward variables P*(*q*_*t *_= *i*, *O*_1, ...,*t*_|*θ*^*m*^) for 1 ≤ *t *≤ *T *iteratively using the forward algorithm [18] for all *i*.

(b) Sample $q_{T}^{m}\sim P\left(q_{T},O|\theta^{m}\right)$.

(c) Sample the state sequence *Q*^*m *^in a backward fashion for *T *>*t *≥ 1.

$$\begin{array}{cc} q_{t}^{m} & \sim P(q_{t}^{m}|q_{t+1}^{m},O,\theta^{m})\\ & \propto P(q_{t}^{m},O_{1},\ldots ,t|\theta^{m})a_{q_{t}^{m}}{,}_{q_{t+1}^{m}}. \end{array}$$

(d) Sample,

$$\begin{array}{ll} \theta^{m+1}\sim & \text{PriorDistribution}\left(H,O,Q^{m},\theta^{m}\right)\;\\ & \left[H=\text{Set}\;\text{of}\;\text{hyperparameters}\right].\;\\ & \; \end{array}$$

Despite its fast convergence, FBG-sampling takes *O*(*MTN*^2^) time for *M *iterations. For long observation sequences with millions of observations, as they are common in biological applications, realistic values for M and N make FGB-sampling intractable. In the next section we discuss how FBG-sampling can be approximated to improve the running time to *O*(γ*MTN*^2^), where γ is the compression ratio of the observation sequence, while maintaining good statistical properties. We refer to our sampling technique as *approximate sampling*.

### Approximate sampling

Through application of a modified kd-tree algorithm (details below), we compress the observation sequence *O *= *o*_1_, ..., *o*_*T *_into $O^{\prime }=o_{1}^{\prime },\ldots ,o_{T^{\prime }}^{\prime }$, cf. Figure 1 (middle), and store precomputed first moment, second moment, and the number of observations compressed into block $o_{t}^{\prime }$ for all *i *≤ *T'*. In subsequent MCMC iterations we assume that *observations compressed in a block *$o_{t}^{\prime }$* arise from the same underlying state*. In other words we *ignore the contribution of the state paths that do not go through the same state for *$o_{t}^{\prime }$. By ignoring those state paths, we refer to them as *weak state paths*, when computing forward-variables and by reusing pre-computed statistics we are able to accelerate sampling.

At first ignoring weak state paths may sound like a very crude approximation for computing *forward variables*. But in many applications this is certainly not true. We demonstrate with a symmetric Gaussian HMM that the *weak state path *assumption is a fairly realistic approximation and leads to faster sampling. We define a symmetric HMM λ = (*A*, *B*, π) with *N *states *s*_1_, ..., *s*_*N*_, where we set self-transition probability *a*_*ii *_= *t *and non-self-transition probability $a_{ij}=\frac{1-t}{N-1}$ for 1 ≤ *i *≠ *j *≤ *N*, and *B *= {(*μ*_1_, *σ*^2^), ..., (*μ*_*N*_, *σ*^2^)}. Given a sequence of observations *O *(assumed to be generated by λ) and its compressed form *O' *we describe an important lemma and some remarks below.

**Lemma 1**. Let $O^{i-1}=o_{1},\ldots ,o_{i-1},o^{\prime }=o_{i},\ldots ,o_{i+n-1},o_{min}^{\prime }=\underset{o_{i}\in o^{\prime }}{\min}o_{l},o_{max}^{\prime }=\underset{o_{i}\in o^{\prime }}{\max}o_{l},d=\underset{j\neq k}{\min}|\mu_{j}-\mu_{k}|$ and $\frac{P\left(q_{i}|O^{i-1}\right)}{P\left(q_{i}=s_{x}|O^{i-1}\right)}\leq \alpha$. Assuming there exists a state *s*_*x *_s.t. $\tau =\min\left({o^{\prime }}_{min}-\frac{\mu_{s_{x-1}}+\mu_{s_{x}}}{2},\frac{\mu_{s_{x}}+\mu_{s_{x+1}}}{2}-{o^{\prime }}_{max}\right)\geq 0$, we can show that $\frac{\underset{\left(q_{i},\ldots ,q_{i+n-1}\right)\in S^{n}}{\sum }P\left(q_{i},\ldots ,q_{i+n-1},o^{\prime }|O^{i-1}\right)}{\underset{s\in S}{\sum }P\left(q_{i}=\ldots =q_{i+n-1}=s,o^{\prime }|O^{i-1}\right)}\leq \alpha \left({\left(1+rc\right)}^{n-1}+\left(N-1\right)c^{\frac{2n}{N}}{\left(1+r\right)}^{n-1}\right)$, where $r=\frac{1-t}{t}$ and $c=e^{-\frac{dx}{2\sigma^{2}}}$.

*Proof*. See Appendix.

**Remark 1 **For realistic values of *τ*, *t*, and *n*, the contribution from ignored weak state paths, which we call *ϵ*, can be very small. If *ϵ *≪ 1, ignoring weak state paths will not introduce large errors in the computation. For the 2-state example in *Section: Results*, where *t *= 0.9, *d *= 1, and *σ*^2 ^= 0.1, *ϵ *is at most $\frac{1}{3}$ for a block length *n *≤ 10 if we assume *τ *> 0.25 and *α *= 1. If *τ *is much larger and consequently $c^{\frac{2n}{N}}$ is much smaller, we can roughly say that *n *can be as large as 1 + log_1+__*rc*_(1 + *ϵ*) in a symmetric Gaussian HMM.

**Remark 2 **We often encounter situations where *P*(*q*_*i *_= *s*_*x*_|*O*^*i*^^-1^) ≫ *P*(*q*_*i *_≠ *s*_*x*_|*O*^*i*^^-1^). Even though it is not exploited in the lemma (*α *being greater than or equal to 1), as a consequence of this, the observation sequence can be compressed into larger blocks keeping *ϵ *small in practice.

From the above lemma and the remarks we see that the longer blocks created by an algorithm should concentrate around the means of the Gaussian distributions. While a brute force algorithm looks at local information, a *kd*-tree like algorithm alternately looks at both dimensions and utilizes global information like the density of data points (around the means data concentration should be high) to create better quality blocks. We use a modified *kd*-tree based algorithm to find such blocks and discuss the details below.

#### kd-tree Based Sequence Compression

Given a starting *width *parameter *w *we create a list of nodes from the observation sequence *O *= *o*_1_, ..., *o*_*T *_using the following steps.

1. Let *O' *= *ϕ *be the starting list, *δ *= 1.25 (picked empirically), level *L *= 1, and dimension *d *= 1.

2. If $|\underset{o_{i}\in O}{\max}\left(o_{i}\right)-\underset{o_{i}\in O}{\max}\left(o_{i}\right)|<\frac{w}{\delta^{L}}$ or |*O*| = 1, create a node storing the first and second moment of the observations in *O*, append it to *O'*, and then go to the end step. Otherwise, go to the next step.

3. If *d *= 1, find *o*_*m*_, the median value of the observations in *O*. Partition *O *into maximal set of consecutive observations *O*^1^, ..., *O*^*i*^, ..., *O*^*p *^such that $\forall_{o\in O^{i}}o\leq o_{m}$ or $\forall_{o\in O^{t}}o\geq o_{m}$. For each *O*^*i*^, go to step 2 with new input set *O*^*i*^, level *L *+ 1, and *d *= 0.

4. If *d *= 0, divide the input set *O *into two parts *O*^*l *^= *o*_1_, ..., *o*_*i *_and *O*^*r *^= *o*_i+1_, ..., *o*_|*O*| _such that $\left|o_{i}-o_{i+1}|\;\geq \underset{j}{\max}|o_{j}-o_{j+1}\right|$. Then for each set *O*^*l *^and *O*^*r*^, go to step 2 keeping the level value *L *unchanged, and setting *d *= 1.

5. End step.

In the above recursive algorithm, *w *states the initial width, *δ *controls the rate of width shrinking in successive levels of the iterations, and *O' *accumulates the compressed blocks of observations. The current iteration level *L*, the current dimension *d*, and the current input set *O *are local variables in the recursive algorithm. Notice that we start with an empty list *O' *and at the end of the recursive procedure *O' *contains an ordered list of compressed observations. To gain further compression of the sequence, we sequentially go through the blocks of *O' *and combine consecutive blocks if the distance between their means is less than *w*. We also combine three consecutive blocks if the outer blocks satisfy this condition and the inner block has only one observation. In step 3 of the above algorithm, the input set is divided into two subsets and each subset contains half of the elements from the original set. Consequently, the height of the recursion tree is at most 2log*T *and the running time of the above algorithm is *O*(*T *log *T*). This overhead is negligible compared to the time that it takes to run *M *iterations of MCMC sampling.

#### Width Parameter Selection

For increasing values of *w *the average block size increases exponentially in the above *kd*-tree based compression. As a result, the compression ratio $\gamma =\frac{T^{\prime }}{T}$ plotted as a function of *w*, has a *knee *which can inform the choice of *w*. Moreover, methods originally developed to find the optimal numbers of clusters in clustering can be used to find the knee of such a curve automatically. In particular, we use the L-method [27] which finds the knee as the intersection of two straight lines fitted to the compression curve.

#### Fast Approximate Sampling

Given the compressed input sequence $O^{\prime }=o_{1}^{\prime },o_{2}^{\prime },\ldots ,o_{T}^{\prime }$ computing forward variables and subsequent sampling is a straightforward modification of the uncompressed case. In particular we make the following two changes to the FBG-sampling algorithm.

1. Modified forward variables at positions $t_{*}=\overset{t}{\underset{i=1}{\sum }}|{o^{\prime }}_{i}|$ are computed using the following formula,

$$\begin{array}{c} P\left(qt_{*}=i,{O\prime }_{1},\ldots ,t|\theta \right)\\ =\underset{1\leq j\leq N}{\sum }P\left(qt_{*}-|{o\prime }_{t}|\;=j,{O\prime }_{1,\ldots ,t-1}|\theta \right)a_{ji}\\ \;\;\;\;\underset{\begin{array}{c} \text{constant time computation}\\ \text{using precomputed statistics} \end{array}}{\underset{︸}{a_{ii}^{|{o\prime }_{t}|-1}\underset{o_{k}\in {o\prime }_{t}}{\prod }P\left(o_{k}|\mu_{i},\sigma_{i}\right).}} \end{array}$$

2. After sampling the state sequence, parameters are updated ignoring non-self transitions between consecutive observations in $o_{t}^{\prime }$.

Clearly, each iteration of approximate sampling takes *O*(*T' N*^2^) resulting in $\frac{T}{T^{\prime }}$ times speed up.

## Results

We evaluate FBG-sampling and approximate sampling in three different settings. First, its effectiveness is verified for a simple two state model. Then, we test on simulated ArrayCGH data which is the accepted standard for method evaluation [28]. Finally, we report findings from an analysis of Mantle Cell Lymphoma (MCL) cell lines [29], Corriel cell lines [30], GBM datasets [31], and high resolution SNP arrays [13,32]. For biological data, if multiple chromosomes are present, we use pooling [25] across chromosomes, which does not allow transition between different chromosomes but assumes model parameters to be identical across chromosomes. Throughout this section we define *σ*_*D *_to be the standard deviation of all observations in the dataset. We compress the dataset with increasing values of *w *= 0.25*σ*_*D*_, 0.5*σ*_*D*_, 0.75*σ*_*D*_, .... For evaluation we consider the experiments as two class problems: aberrant clones belong to the positive class and normal clones belong to the negative class. When ground truth labels of a dataset are available we report F1-measure, recall, and precision for the experiment. With *tp*, *fp*, *tn*, *fn *we denote the number of true and false positives and true and false negatives respectively. Recall is defined as $\frac{tp}{tp+fn}$, precision as $\frac{tp}{tp+fp}$, and F1-measure as $\frac{2\times recall\times precision}{recall+precision}$. Experiments were run with a Python implementation on a Linux machine with 1.6 GHz Intel Core 2 Duo processor and 2 GB memory. For Expectation Maximization (EM), we use the Baum-Welch algorithm from the GHMM package which is implemented in C and considerably faster than a Python implementation.

### Synthetic Data

#### 2-State HMM

We define a HMM λ_2__*ST *_= (*A*, *B*, *π*) with $A=\left[\left[0.9,0.1\right],\left[0.1,0.9\right]\right],B=\left[\left(0,0.1\right),\left(1,0.1\right)\right],\pi =\left[\frac{1}{2},\frac{1}{2}\right]$. From λ_2__*ST *_we sample an observation sequence *O *= *o*_1_, ..., *o*_10,000_, and run MCMC for *M *= 100 steps with hyperparameter values ${\tilde{\mu }}_{1:2}=0,1$ for the prior mean on *μ*, ${\tilde{\sigma }}_{1:2}=0.5,0.5$ for the prior variance on *μ*, *a*_1:2 _= 4, 4 for the shape of Gamma prior on σ^-2^, *b*_1:2 _= 1, 1 for the rate of Gamma prior on *σ*^-2^, *δ*^*π *^= 1, 1 for the Dirichlet prior on the initial distribution *π*, and $\delta_{1:2}^{At}=1,1$ for the Dirichlet prior on row *i *of transition matrix *A*.

**Figure 2:**
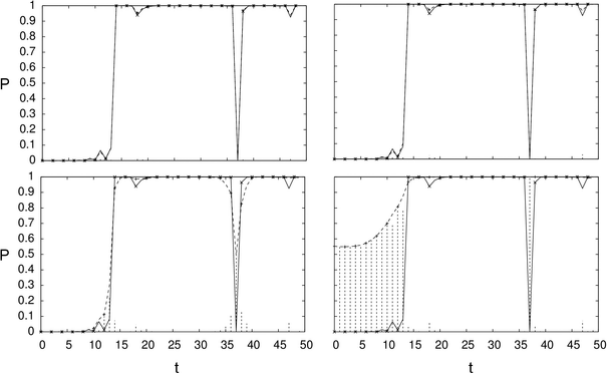
**Simulated data: approximate posterior**. We show the posterior probability of state 1 (y-axis) for first fifty observations (x-axis) with *w *= 0.5*σ*_*D *_(top left), 1.0*σ*_*D *_(top right), 1.5*σ*_*D *_(bottom left), and 2.0*σ*_*D *_(bottom right). The true posterior is shown as a solid line, the approximate posterior as a dashed line, and their absolute difference is shown in dashed vertical lines.

After *M *iterations, we compare the posterior probabilities $P\left(q_{t}=i|O,\theta_{FBG}^{M}\right)$ and $P\left(q_{t}=i|O,\theta_{A}^{M}\right)$, where $\theta_{FBG}^{M}$ and $\theta_{A}^{M}$ are *M*-th parameter samples of FBG-sampling and approximate sampling. Figure 2 shows that the posterior probability of being in state 1 for each position can be approximated fairly well even for large values of *w*. The average posterior error $\tilde{P}=\frac{1}{2T}\sum_{t}\sum_{i}|P\left(q_{t}=i|\theta^{M},O\right)-P\left(q_{t}=i|\theta^{true},O\right)|$ reflects the same fact in Table 1. Similarly, we compute the Viterbi paths and report total number of mismatches between them along with the likelihoods in Table 1.

**Table 1:** We show the average posterior error $\tilde{P}=\frac{1}{2T}\sum_{t}\sum_{i}|P\left(q_{t}=i|\theta^{M},O\right)-P\left(q_{t}=i|\theta^{true},O\right)|$ and total number of mismatches between the two Viterbi paths , generated by models with parameters *θ*^*true *^and *θ*^*M*^

Method	*w *(in *σ*_*D*_)	$\tilde{P}$		Likelihood	Time(in sec)	Speed up
	0.25	0.001	3	-5470	74	1.2
	0.50	0.001	3	-5475	61	1.4
	0.75	0.002	6	-5469	35	2.4
Approx	1.00	0.004	22	-5478	21	4.2
	1.25	0.012	81	-5588	13	6.5
	1.50	0.054	410	-6576	8	10.4
	1.75	0.219	2345	-8230	4	20.1
	2.00	0.248	2857	-8492	3	34.1

FBG	...	0.003	12	-5471	87	1.0

True	...	*...*		-5470		*...*

#### Simulation from Genetic Template

We use 500 simulated datasets published in [28]. Each dataset has 20 chromosomes and 100 clones per chromosome for a total of 2,000 clones per dataset. A four-state HMM predicts the aberrant regions--loss defined as state *S*_1 _and gain defined as state *S*_3 _or *S*_4_. The neutral region is modeled as state *S*_2_. We put an ordering constraint on the means, *μ*_1 _<*μ*_2 _<*μ*_3 _<*μ*_4_, to prevent label switching of the states [17]. Hyperparameter choices follow [25] and are ${\tilde{\mu }}_{1:4}=-0.5,0,0.58,1$ for the prior mean on *μ*, ${\tilde{\sigma }}_{1:4}=0.5,0.001,1.0,1.0$ for the prior variance on *μ*, *a*_1:4 _= 10,100, 5, 5 for the shape of gamma prior on *σ*^-2^, and $b_{1:4}=\delta^{\pi }=\delta_{1:4}^{A_{t}}=1,1,1,1$ for the rate of gamma prior on σ^-2^, the Dirichlet prior on initial distribution *π*, and the Dirichlet prior on row *i *of transition matrix *A*, respectively.

**Figure 3:**
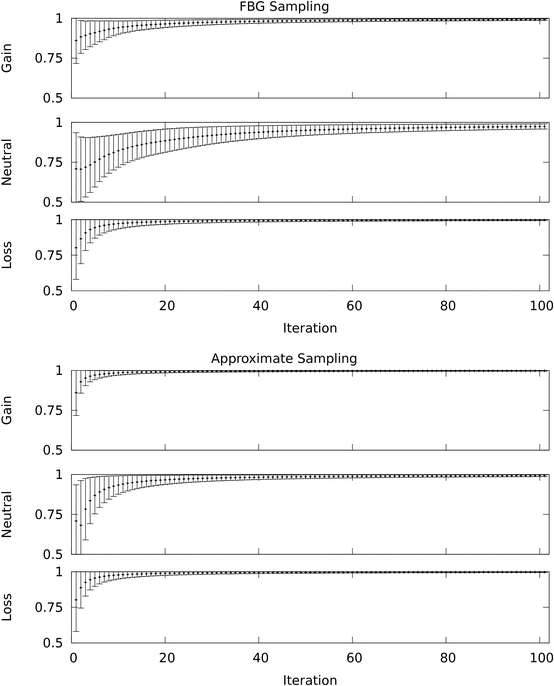
**MCMC convergence**. The convergence of posterior probabilities for loss, neutral, and gain of three representative probes--probe 1658, probe 1512, and probe 447 respectively--from the simulated dataset 63 are shown. For each probe, at first, the posterior probability of the corresponding HMM state, given the sampled parameters from the current MCMC iteration, is computed. The time-average of these posterior probabilities, starting from the first iteration to the current iteration, approximates the posterior of the HMM state given the data. The mean of the posterior probabilities over 10 MCMC chains are shown with error bars (mean ± one standard deviation)--loss probe in the bottom row, neutral probe in the middle, and the gain probe in the top row. The top figures show the outcomes of FBG sampling and the bottom figures show the outcomes of approximate sampling. Note that the reduction in standard deviation suggests that approximate sampling converges quicker than FBG sampling for these probes.

Table 2 shows the mean and standard deviation of F1-measure, recall, and precision over the 500 datasets for FBG-sampling, approximate sampling, and Expectation Maximization (EM) with the ground truth provided by [28]. Even for this collection of relatively small datasets we see a 10-fold speed up. For each dataset we run FBG and approximate sampling for *M *= 100 steps (we have visually monitored the parameters and noticed convergence within 50 steps, see Figure 3 for a representative example). The last 10 samples are used to compute 10 samples of the posteriors for each state and for each position in the observation sequence. Subsequently, aberrant regions are predicted based on the average of those distributions. We report the speed-up of approximate vs. FBG sampling based on the time it takes to compress the sequence and run *M *steps of MCMC. For one individual dataset EM requires 58 seconds on average, which allows for a total of 800-1000 repetitions from randomized points sampled from the prior distributions in the time needed for FBG sampling. Each run continues until the likelihood converges and the best model based on likelihood is selected. Aberrant regions are predicted and compared against the ground truth based on the Viterbi path. We report the mean and standard deviation of F1-measure, recall, and precision over the results of EM on 500 datasets.

**Table 2:** EM, FBG-sampling, and approximate sampling results for simulated, HBL-2, and Corriel dataset are shown here

Dataset	Method	*w*	F1-measure	Recall	Precision	Time	Compression	Speed-up	Likelihood
		0.50	**0.97 **± 0.04	0.96 ± 0.07	**0.98 **± 0.02	27	0.387	2.2	
		0.75	**0.97 **± 0.04	0.96 ± 0.06	**0.98 **± 0.03	16	0.195	3.7	
		1.00	**0.97 **± 0.05	0.95 ± 0.07	**0.98 **± 0.03	10	0.097	5.9	
	Approx	*1.25*	0.96 ± 0.06	0.94 ± 0.09	**0.98 **± 0.03	7	0.050	8.8	
Simulated		1.50	0.94 ± 0.09	0.92 ± 0.12	0.97 ± 0.07	5	0.031	11.3	
		1.75	0.91 ± 0.15	0.89 ± 0.18	0.96 ± 0.12	5	0.023	12.2	
		2.00	0.86 ± 0.19	0.85 ± 0.21	0.92 ± 0.19	5	0.018	12.2	
	
	FBG	...	**0.97 **± 0.04	0.96 ± 0.05	**0.98 **± 0.03	58	...	1.0	
	
	EM prior, ML	...	0.96 ± 0.09	**0.97 **± 0.04	0.96 ± 0.11	58	...	...	

		*1.0*	**0.85 **± 0.00	0.83 ± 0.00	**0.88 **± 0.00	72	0.078	11.3	
		2.0	**0.87 **± 0.00	0.83 ± 0.00	**0.91 **± 0.00	21	0.018	39.3	
		3.0	**0.89 **± 0.00	0.83 ± 0.00	**0.95 **± 0.00	13	0.006	61.8	
	Approx	4.0	**0.84 **± 0.08	0.77 ± 0.11	**0.95 **± 0.01	13	0.003	61.9	
		5.0	0.71 ± 0.17	0.60 ± 0.22	**0.95 **± 0.01	13	0.002	64.8	
		6.0	0.79 ± 0.07	0.69 ± 0.10	**0.96 **± 0.01	14	0.002	59.3	
		7.0	0.76 ± 0.08	0.64 ± 0.11	**0.93 **± 0.01	13	0.001	61.4	
	
HBL-2	FBG	...	0.82 ± 0.00	0.84 ± 0.00	0.80 ± 0.00	810	...	1.0	
	
	EM prior, ML	...	0.65	**0.90**	0.50	810	...	...	15158
	
	EM prior, best	...	0.85	0.84	0.86	810	...	...	9616
	
	EM prior, mean	...	0.76 ± 0.09	0.86 ± 0.03	0.68 ± 0.12	810	...	...	13744
	
	EM unif, ML	...	0.64	0.90	0.50	810	...	...	15159
	
	EM unif, best	...	0.86	0.84	0.88	810	...	...	9136
	
	EM unif, mean	...	0.54 ± 0.24	0.77 ± 0.21	0.46 ± 0.27	810	...	...	13457

GM05296	Approx	2.0	**0.96 **± 0.00	**1.00 **± 0.00	**0.93 **± 0.01	3	0.027	13.0	
	
	FBG	...	0.89 ± 0.01	**1.00 **± 0.00	0.81 ± 0.01	40	...	1.0	

GM00143	Approx	2.0	**0.98 **± 0.00	**1.00 **± 0.00	**0.96 **± 0.00	3	0.027	13.8	
	
	FBG	...	0.89 ± 0.24	**1.00 **± 0.00	0.86 ± 0.26	40	...	1.0	

Approximate sampling results are reported for different choices of *w*. The *w *value which is closest to the one estimated by the L-method is shown in italic. Width *w *is reported in σ_*D *_of the corresponding dataset, time is reported in seconds, and compression is defined as $\frac{T^{\prime }}{T}$. For HBL-2, the initial parameter values for EM are sampled from the prior or uniform distributions, and the average (mean), most likely (ML), and best (in terms of F1-measure) performances along with likelihoods are reported.

### Biological Data

#### Mantle Cell Lymphoma (MCL)

**Figure 4:**
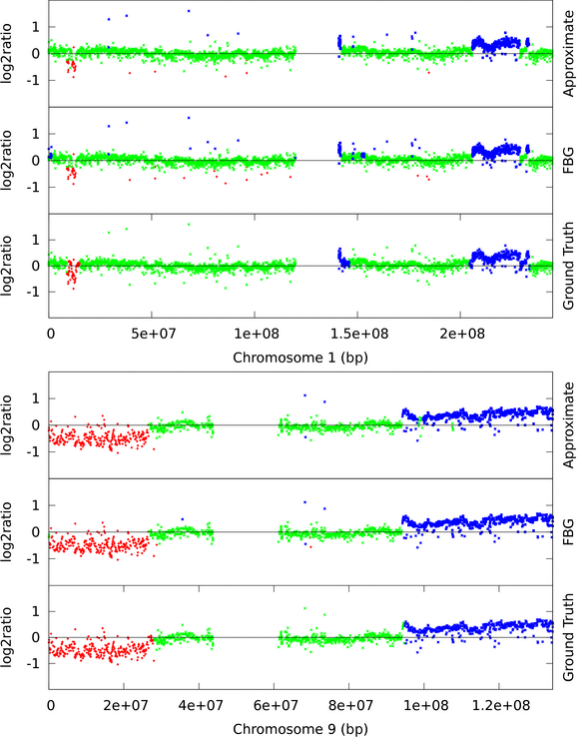
**HBL-2: chromosome 1 and 9**. We contrast the ground truth and the segmentations produced by FBG-sampling and approximate sampling. For approximate sampling *w *was set to the value recommended by the L-method. Here, clones predicted as loss are shown in red, normal clones in green, and gain in blue. The figure at the top shows chromosome 1 and the bottom figure shows chromosome 9.

De Leeuw and colleagues identified recurrent variations across cell lines using ArrayCGH data of MCL cell lines [29]. Out of the eight cell lines [29] HBL-2 was fully annotated with marked gain and loss regions in the autosomes. This dataset contains about 30,000 data points (combining all the autosomes). We have used a four-state HMM for predicting aberrant regions. State 1 represents copy number loss, state 2 represents normal copy number, state 3 represents single copy gain, and state 4 multiple gain. For HBL-2 we report the F1-measure, recall, precision and speed-up. Similar to the synthetic case we put an ordering constraint on the means, *μ*_1 _<*μ*_2 _<*μ*_3 _<*μ*_4_. Hyperparameter choices follow [25] and are same as for the simulation from genetic template, except for ${\tilde{\sigma }}_{1:4}=0.2,0.1,0.2,0.2$, the prior variance on *μ*, and *a*_1:4 _= 15, 20, 10, 10, the shape of gamma prior on *σ*^-2^. Settings for FBG-sampling and approximate sampling are identical to the simulated case with one exception; for each simulated dataset sampling methods run once and we report the average and standard deviation over 500 datasets, but for HBL-2 we let them run 10 times and report the average and standard deviation of these 10 F1-measures, recalls, and precisions in Table 2. Each EM run starts with the initial parameter values sampled either from the prior distributions, or from uniform distributions, and continues until the likelihood value converges. We report the performance of the most likely model (which is the preferred criteria to select a model), the likelihood of the best model based on F1-measure, and the average and standard deviation of F1-measures, recalls, and precisions of all the models generated by EM. As representative examples, we also plot the segmentation of chromosome 1 and 9 computed by FBG-sampling and approximate sampling along with the ground truth labels in Figure 4.

#### Corriel

Corriel cell lines were used by Snijders *et al*. [30] and are widely considered a gold standard in ArrayCGH data analysis. This dataset is smaller and, in fact, fairly easy compared to the MCL cell lines. For the Corriel cell line we use a 4-state HMM and report the results for GM05296 and GM00143 in Table 2. Again, approximate sampling performs competitively while achieving more than a 10-fold speed-up. Hyperparameter choices follow [24].

#### GBM

**Figure 5:**
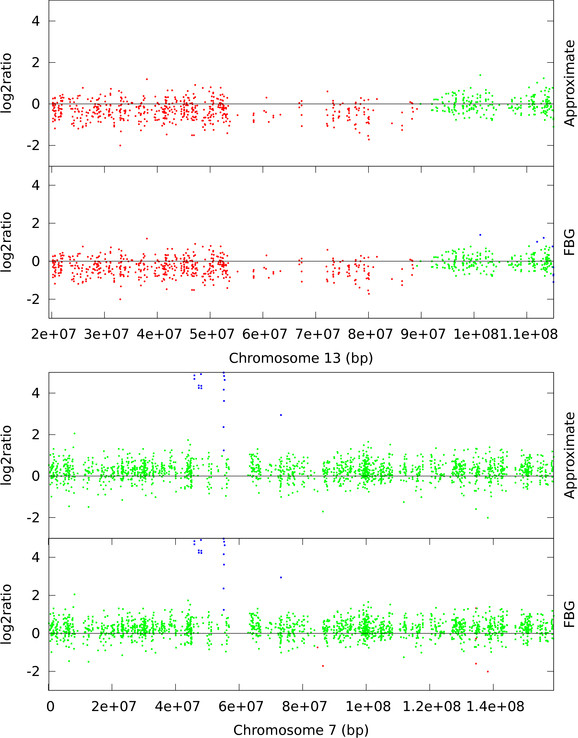
**GBM: chromosome 7 (GBM29) and chromosome 13 (GBM31)**. Loss (red), normal (green), and gain (blue) clones are identified using FBG-sampling and approximate sampling. For approximate sampling *w *= 1.5*σ*_*D *_is used, which was recommended by the L-method.

The glioma data from Bredel *et al*. [31] has previously been used to analyze the performance of CNV detection methods [9,33]. According to [33], GBM datasets are noisy but contains a mixture of aberrant regions with different width and amplitude. In particular, chromosome 13 of GBM31 is reported to have low amplitude loss in p-arm and chromosome 7 of GBM29 is reported to have high amplitude gains near the EGFR locus by previous studies [9,33]. The segmentation of these two chromosomes are shown in Figure 5. Although [33] reports that EM based HMM failed to detect these aberrations we see that Bayesian HMM has successfully detected both the gain in chromosome 7 and the loss in chromosome 13. For this dataset, we use a 3-state HMM with non-informative hyperparameters, ${\tilde{\mu }}_{1:3}=-\frac{\sigma_{D}}{2},0,\frac{\sigma_{D}}{2}$ for the prior mean on *μ*, ${\tilde{\sigma }}_{1:3}=0.2,\;0.1,\;0.2$ for the prior variance on *μ*, $a_{1:3}=\frac{1}{\sigma_{D}^{2}},\frac{1}{\sigma_{D}^{2}},\frac{1}{\sigma_{D}^{2}}$ for the shape of gamma prior on *σ*^-2^, *δ*^*π *^= 1, 9, 1 for the Dirichlet prior on initial distribution *π*, and $b_{1:3}=\delta_{1:3}^{A_{t}}=1,1,1$ for the rate of gamma prior on σ^-2 ^and the Dirichlet prior on row *i *of transition matrix *A*, respectively, and at the recommended *w *value we see a 10 fold speed-up.

#### SNP Array

**Figure 6:**
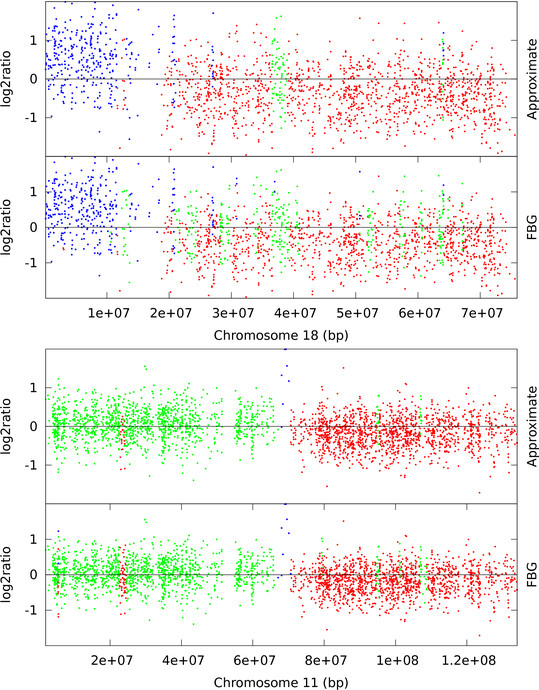
**Affymetrix 100 k SNP array: chromosome 18 of sample 16 and chromosome 11 of sample 33**. Loss (red), normal (green), and gain (blue) clones are identified using FBG-sampling and approximate sampling. For approximate sampling *w *= 1.8*σ*_*D *_is used, which was recommended by the L-method.

**Figure 7:**
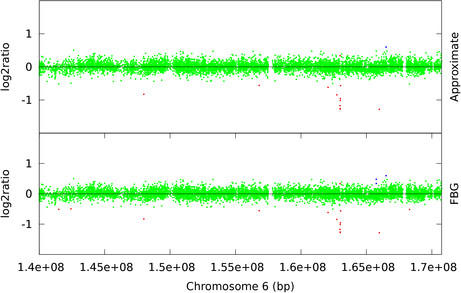
**Illumina HumanMap550 array: chromosome 6 of sample 3**. Loss (red), normal (green), and gain (blue) clones are identified using FBG-sampling and approximate sampling. For approximate sampling *w *= 1.6*σ*_*D *_is used, which was recommended by the L-method.

High-resolution Single Nucleotide Polymorphism (SNP) arrays are capable of detecting smaller CNVs than ArrayCGH. To demonstrate the computational advantage of approximate sampling on SNP arrays we have chosen publicly available Affymetrix 100 k pancreatic cancer datasets from [32] and Illumina HumanHap550 arrays of HapMap individuals which are provided as examples in PennCNV [13]. An Affymetrix 100 k dataset consists of two different arrays each with ≈ 60, 000 SNP markers and, in total, 10^5 ^data points per sample. On the other hand, the Illumina HumanHap550 array has around half a million SNP markers. We have applied FBG-sampling and approximate sampling with *w *= 1.8*σ*_*D*_, the recommended value by the L-method, to the sample datasets from Harada *et al*. [32] and found that the computational speed-up is 22-fold (100 runs of FBG-sampling takes 1844 seconds). Both sampling approaches mostly agree on their predictions but they, specially FBG-sampling, detect several more CNVs than previously identified [32]. For example, the high amplification in chromosome 11 (sample 33) is successfully identified by all methods but in chromosome 18 (sample 16) the sampling algorithms find a few normal regions previously predicted [32] as loss using the CNAG tool [34] (see Figure 6). One possible reason for these differences is that while we use 269 HapMap samples as reference they use 12 unpublished normal samples as reference. Similarly, we have tested our method with 2.0*σ*_*D *_≤ *w *≤ 3.0*σ*_*D *_against Illumina HumanHap samples and observed 70 to 90 fold speed-up in computations (100 runs of FBG-sampling takes 9693 seconds). These samples are taken from apparently healthy individuals and contain very few CNVs. As expected, both sampling algorithms' predictions are nearly identical and they seem to predict extreme outliers as aberrant markers. In contrast, PennCNV [13] does not report CNVs which are covered by less than 3 SNPs, thus suppressing the outliers as normal. We plot a typical region (from 1.4*e *+ 08bp to 1.7*e *+ 08bp) of chromosome 6 from sample 3 (ID 99HI0700A) in Figure 7.

To set hyperparameters we follow the default parameters of the HMM used in PennCNV [13]. We have observed that HMMs for large arrays are particularly sensitive to the self-transition probabilities (which is also reflected in the default parameter values of the HMM used in PennCNV). Hence, hyperparameters were set to reflect the choice of high self-transition probability for each state--we set $\delta_{1:3}^{A_{t}}=\alpha_{i1}l,\alpha_{i2}l,\alpha_{i3}l$, the Dirichlet prior on row *i *of transition matrix *A*, where *l *= 5000, α_*ii *_is 0.99 for *i *= 2, 0.95 for *i *≠ 2, $\alpha_{ij}=\frac{1-\alpha_{ii}}{2}$ for *i *≠ *j*. Other hyperparameters for the 3-state HMM were set such that the expected values of prior distributions match the default values for PennCNV. In particular, they were ${\tilde{\mu }}_{1:3}=10.66,0,0.54$ for the prior mean on *μ*, ${\tilde{\sigma }}_{1:3}=0.001,0.001,0.001$ for the prior variance on *μ*, *a*_1:3 _= 12, 30, 25 for the shape of gamma prior on *σ*^-2^, *b*_1:3 _= 1, 1, 1 for the rate of gamma prior on *σ*^-2^, and *δ*^*π *^= 1, 9, 1 for the Dirichlet prior on initial distribution *π*, respectively.

### Discussion

#### EM vs. MCMC

As already a 4-state Gaussian HMM has 23 free parameters applying EM is often difficult due to the existence of multiple local optima and the local convergence of EM. Consequently, the estimate has to be repeated many times with randomly initialized parameter values to find the most likely model. It should also be noted that not necessarily the model maximizing the likelihood exhibits the best performance in classifying aberrations 2. Additionally, applying any constraint in an EM settings, i.e. ordering of the state means, is harder than in MCMC. EM also produces inferior results on datasets exhibiting class imbalance, for example where one type of aberrations (observations for one HMM state) are rare or missing, while MCMC can overcome this problem using informative priors. In Table 2 we see that MCMC sampling performs better than EM on real biological data which is consistent with prior reports from Guha [24] and Shah [25] who also report difficulties with EM and better MCMC performances. In particular, for HBL-2 we observe that the best model in terms of F1-measure--which is not available in *de novo *analysis--is not the most likely model and the most likely model has very low precision and, consequently, worse F1-measure than MCMC sampling. On the simulated datasets, EM performs poorly if the dataset is imbalanced. As a result we observe slightly worse standard deviation for the precisions and F1-measures computed by EM in Table 2. We also notice from the confusion matrix of three classes--loss, neutral, and gain--that even though the mean F1-measure, recall, and precision (defined on two classes, neutral and aberrant) are high, due to label switching [17], EM does not distinguish gain from loss, and vice versa, very well (see Table 3). By re-ordering the already learned state means the label switching problem can be addressed, but that increases misclassification rate due to state collapsing as the parameter values, specially means of the Gaussians, become almost identical [17]. In contrast, Bayesian methods cope with class imbalance problem by applying order constraints. Moreover, using McNemar's test [35] on the combined result of the 500 datasets we have verified that the predictions based on EM are significantly different from the predictions made by Bayesian methods with p-values being less than 0.001 in both cases.

**Table 3:** Confusion matrices showing the proportion of accurate predictions based on EM, FBG-sampling, and approximate sampling methods on 500 simulated datasets

			Truth	
		
		Loss	Neutral	Gain
	Loss	0.855	0.071	0.074
EM	Neutral	0.001	0.996	0.003
	Gain	0.190	0.087	0.723

	Loss	0.980	0.020	0.000
FBG	Neutral	0.002	0.995	0.003
	Gain	0.002	0.020	0.973

	Loss	0.981	0.019	0.000
Approx. (*w *= 1.25*σ*_*D*_)	Neutral	0.002	0.993	0.005
	Gain	0.009	0.022	0.969

#### FBG vs. Approximate Sampling

In an ideal setting, like the 2-state HMM example, approximate sampling closely mimics the performance of FBG sampling up to moderate compression level. For the simulated and real dataset approximate sampling's performance is comparable to FBG's while achieving a speed-up of 10 or larger. We also observe that for higher compression levels approximate sampling reports small number of aberrant clones, which results in small *tp *and *fp *values, but large *fn *value. As a result, we see low recall and high precision rate when the compression level is too high for a particular dataset (see the rows with *w *≥ 4.0*σ*_*D *_for HBL-2 in Table 2).

From Figures 4, 5, 6, and 7 we observe that segmentations by both sampling methods are almost identical at the recommended width *w *value. In case of HBL-2, they differ from the ground truth in some places. They predict a few extra gain regions and outliers are generally predicted as gains. We, as well as Shah *et al*. [25], have noticed that the HBL-2 dataset has many outliers, and the variance of emission distribution of gain state 4 converges to a high value which tries to explain the outliers. In contrast, GBM has fewer outliers (see Figure 5) and approximate sampling seems robust to those outliers. As the compression algorithm forces possible outliers to be included in a compressed block, it is robust to moderate frequencies of outliers.

#### Width Parameter

**Figure 8:**
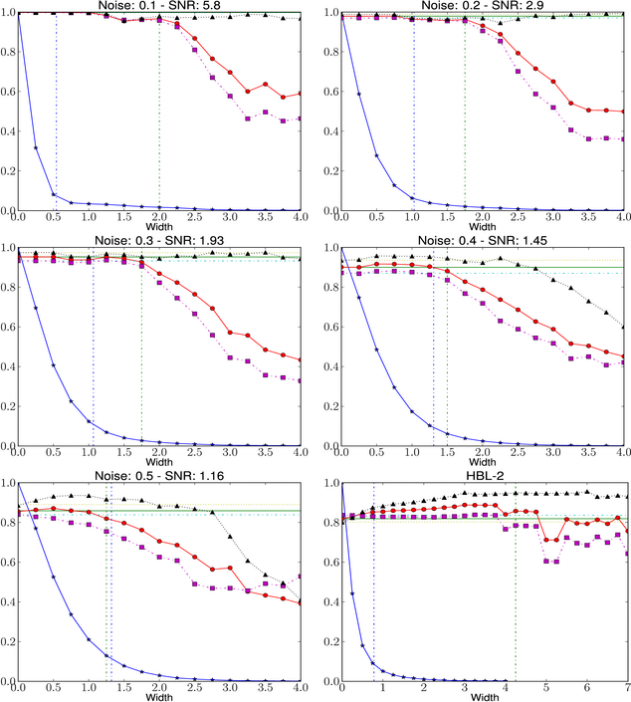
**Effect of width parameter**. F1-measure (red, circle), recall (violet, square), and precision (black, triangle) of approximate sampling over HBL-2 and five synthetic datasets of varying noise levels are shown. For comparison, F1-measure (green, solid), recall (cyan, dashed dot), and precision (olive, dotted) of FBG-sampling are also shown as horizontal lines. For width values 0.0*σ*_*D*_, ..., 4.0*σ*_*D *_compression ratio $\gamma =\frac{T^{\prime }}{T}$ is shown as blue line with stars. Knee is predicted using L-method and shown as a vertical line (blue, dashed dot). Vertical line (green, dashed dot) showing best width is selected by visual inspection.

By varying the width parameter *w *we can control the compression ratio γ and the speed-up achieved by approximate sampling. But from Table 1 and 2, and Lemma 1 it is also clear that by setting a large value one can get unfavorable results. We have analyzed the effect of different *w *values using a synthetic dataset with a controlled level of noise following [36]. Each dataset has three chromosomes with total probe counts 500, 750, and 1000. Ten aberrant regions of size 11-20 probes, randomly assigned gain or loss, are inserted in random positions of the 500 probe chromosome. Similarly, 15 aberrant regions of size 11-25 probes, randomly assigned gain or loss, are inserted into larger chromosomes. A noise component *N*(0, *σ*) is added to the theoretical log-ratios -1,0,0.58 (loss, neutral, and gain respectively) to model the data. For a set of noise levels, *σ *ranging from 0.1 to 0.5, 50 synthetic datasets are generated. We use a 3-state HMM with width parameter values in the range 0*σ*_*D*_, ..., 4*σ*_*D *_(where *σ*_*D *_is the standard deviation of the dataset). Signal-to-noise ratio (SNR) is defined as $\frac{0.58}{\sigma }$. In Figure 8 we plot the mean compression ratio, F1-measure, recall, and precision for 50 synthetic datasets and the real biological data HBL-2. For all noise levels the compression ratio drops exponentially with increasing values of *w*. Ideally, one would like to compress as much as possible without affecting the quality of the predictions. We visually identified a best value for width as the point after which the F1-measure drops substantially. Comparing the knee of the curve with the best value, we notice that while using the knee computed by L-method [27] is a conservative choice for width, in most cases we can still obtain a considerable speed-up.

#### Outliers

Gaussian HMMs are known to be sensitive to outliers which is evident from our results of HBL-2 and SNP arrays. Traditionally, outliers have been handled either by using a mixture distribution as the emission distribution or by preprocessing the data to remove possible outliers or impute more appropriate values. We have observed that a simple local median approach works very well to identify the outliers in a time series of log_2_-ratio values. Although using a mixture distribution or a distribution with fat tails, i.e. Student's-t distribution, is a better choice we lose a significant computational advantage in approximate sampling. For a block of observations *o' *= *o*_*i*_, ..., *o*_*k *_we can compute $\overset{k}{\underset{j=i}{\prod }}P\left(o_{j}|q^{\prime },\theta \right)$ in constant time using precomputed values $\overset{k}{\underset{j=1}{\sum }}o_{j}$ and $\overset{k}{\underset{j=i}{\sum }}o_{j}^{2}$ if the emission distribution is Gaussian. But it is not obvious how we can accomplish this for a more complicated distribution. Another approach, which we prefer in this context, is to use a HMM state with a very wide Gaussian and low self-transition probability to model the outliers. We have observed very good performance in this way. However, as our primary focus is to compare FBG-sampling with approximate sampling we choose to use a simple Gaussian model at the end.

## Conclusions

Analyzing CGH data either from DNA microarrays or next generation sequencing to estimate chromosomal aberrations or investigate copy number variations (CNV), leads to the problem of segmenting sequences of observations which are essentially noisy versions of piecewise-constant functions. For reasons of efficiency, ML or MAP point estimates of HMM parameters combined with the Viterbi-algorithm to compute a most likely sequence of hidden states and thus a segmentation of the input are most popular in practice. This ignores research which clearly demonstrates that Bayesian approaches, where MCMC is used for sampling and thus integrating out model parameters, is more robust with higher recall and higher precision [24]. Additionally, our experiments show that likelihood is not informative with respect to the quality of CNV calls putting the use of ML into question even if the estimation problem could be solved.

We propose a method using approximate sampling to accelerate MCMC for HMMs on such data. Our method constitutes the first use of ideas from spatial index structures for several consecutive observations and approximate computations based on geometric arguments for HMM; the effectiveness of this approach was previously demonstrated for *k*-means clustering, mixture estimation, and fast evaluation of a mixture of Gaussians.

We demonstrate that these very abundant biological CGH datasets, which measure chromosomal aberrations and copy number variations, are consistent with our assumptions of piece-wise constant ground truths, and we are able to achieve speed-ups between 10 and 60 respectively 90, on these biological datasets while maintaining competitive prediction accuracy compared to the state-of-the-art. As datasets with even higher resolution, both from higher density DNA microarrays and next generation sequencing, become available, we believe that the need for precise *and *efficient MCMC techniques will increase. The added precision over popular ML/MAP-based methods is of particular biological relevance as for complex neurodegenerative diseases such as Autism de-novo copy number variations have recently been shown to play a role [37]; a precise and quick analysis on large collectives of patients is desirable.

Applying approximate sampling to multi-dimensional observations--to jointly analyze data sets for recurrent CNVs [38] instead of analyzing individuals and post-processing results--and considering more complicated HMM topologies and observation densities are directions for our future work.

## Authors' contributions

MPM and AS designed the study and wrote the manuscript. MPM implemented approximate sampling and tested it's performance. All authors read and approved the final manuscript.

## Appendix

**Lemma 1**. Let $O^{i-1}=o_{1},\ldots ,o_{i-1},o^{\prime }=o_{i},\ldots ,o_{i+n-1},o_{min}^{\prime }=\underset{o_{i}\in o^{\prime }}{\min}o_{l},o_{max}^{\prime }=\underset{o_{i}\in o^{\prime }}{\max}o_{l},d=\underset{j\neq k}{\min}|\mu_{j}-\mu_{k}|$ and $\frac{P\left(q_{i}|O^{i-1}\right)}{P\left(q_{i}=s_{x}|O^{i-1}\right)}\leq \alpha$. Assuming there exists a state *s*_*x *_s.t. $\tau =\min\left({o^{\prime }}_{min}-\frac{\mu_{s_{x-1}}+\mu_{s_{x}}}{2},\frac{\mu_{s_{x}}+\mu_{s_{x+1}}}{2}-{o^{\prime }}_{max}\right)\geq 0$, we can show that $\frac{\underset{\left(q_{i},\ldots ,q_{i+n-1}\right)\in S^{n}}{\sum }P\left(q_{i},\ldots ,q_{i+n-1},o^{\prime }|O^{i-1}\right)}{\underset{s\in S}{\sum }P\left(q_{i}=\ldots =q_{i+n-1}=s,o^{\prime }|O^{i-1}\right)}\leq \alpha \left({\left(1+rc\right)}^{n-1}+\left(N-1\right)c^{\frac{2n}{N}}{\left(1+r\right)}^{n-1}\right)$, where $r=\frac{1-t}{t}$ and $c=e^{-\frac{dx}{2\sigma^{2}}}$.

*Proof*. Using the assumption on τ, for any position *i *≤ *l *≤ *i *+ *n *- 1, we can argue that,

(1) $$\begin{array}{ll} \frac{e^{-\frac{1}{2}{\left(\frac{o_{l}-\mu_{q_{l}}}{\sigma }\right)}^{2}}}{e^{-\frac{1}{2}{\left(\frac{o_{l}-\mu_{s_{x}}}{\sigma }\right)}^{2}}} & \leq e-\frac{|\mu_{q_{l}}-\mu_{s_{x}}|\tau }{\sigma^{2}}\;\\ & \leq \left\{\begin{array}{cc} 1 & \text{if}\;q_{l}=s_{x},\\ e^{-\frac{d\tau }{\sigma^{2}}} & \text{otherwise}\text{.} \end{array}\right)\;\\ & \; \end{array}$$

For any partial state path *q*_*i*_, . . ., *q*_*i*__+__*n*__-1_,

(2) $$\begin{array}{c} P\left(q_{i},\ldots ,q_{i+n-1},o^{\prime }|O^{i-1}\right)\\ =P\left(q_{i}|O^{i-1}\right)P\left(o_{i}|q_{i},O^{i-1}\right)\\ \overset{i+n-2}{\underset{k=i}{\prod }}a_{q_{k}q_{k+1}}P\left(o_{k+1}|q_{k+1}\right)\\ =P\left(q_{i}|O^{i-1}\right)\frac{e^{-\frac{1}{2}{\left(\frac{o_{i}-\mu q_{i}}{\sigma }\right)}^{2}}}{\sqrt{2\pi \sigma^{2}}}\\ \overset{i+n-2}{\underset{k=i}{\prod }}a_{q_{k}q_{k+1}}\frac{e^{-\frac{1}{2}{\left(\frac{o_{k+1}-\mu_{q_{k+1}}}{\sigma }\right)}^{2}}}{\sqrt{2\pi \sigma^{2}}}. \end{array}$$

We partition *S*^*n*^, the set of all possible partial state paths of length *n*, into *N *subsets $S^{s_{1}}\ldots S^{s_{N}}$ such that, $S^{s_{j}}=\left\{\tilde{S}\in S^{n}:\left(\forall_{s_{l}\neq s_{j}}C\left(\tilde{S},s_{j}\right)>C\left(\tilde{S},s_{l}\right)\right)\vee \left(\left(\forall_{s_{l}\neq s_{j}}C\left(\tilde{S},s_{j}\right)\geq C\left(\tilde{S},s_{l}\right)\right)\wedge {\tilde{S}}_{1}=s_{j}\right)\right\}$ for 1 ≤ *j *≤ *N*, where $C\left(\tilde{S},s\right)=\underset{q_{k}\in \tilde{S}}{\sum }1\left(q_{k}=s\right)$. We again partition $S^{s_{j}}=\cup_{k=0}^{n-1}S_{k}^{s_{j}}$ such that, $S_{k}^{s_{j}}=\left\{\tilde{S}\in S^{s_{j}}:\left(\overset{n-1}{\underset{l=1}{\sum }}1\left({\tilde{S}}_{l}\neq {\tilde{S}}_{l+1}\right)\right)=k\right\}$.

The size of *S*^*n *^can be expressed in terms of total number of non-self-transitions present in a path,

(3) $$\begin{array}{ll} |S^{n}|\; & =N^{n}\;\\ & =N\overset{n-1}{\underset{k=0}{\sum }}\left(\begin{array}{c} n-1\\ k \end{array}\right){\left(N-1\right)}^{k}.\;\\ & \; \end{array}$$

As the sets $S^{s_{j}}$ are equal sized partitions of *S*^*n*^, $|S^{s_{j}}|\;=\overset{n-1}{\underset{k=0}{\sum }}\left(\begin{array}{c} n-1\\ k \end{array}\right){\left(N-1\right)}^{k}$. Also notice that, by definition, the partial state paths in *S*^*n *^with exactly *k *number of non-self-transitions are equally distributed among the subsets $S^{s_{j}}$. As a result, $|S_{k}^{s_{j}}|\;=\left(\begin{array}{c} n-1\\ k \end{array}\right){\left(N-1\right)}^{k}$.

Now we define *S*^[s] ^= {(*q*_*i*_, ..., *q*_*i*__+__*n*__-1_) : (*q*_*i *_= ... = *q*_*i*__+__*n*__-1 _= *s*)}. For the remaining part of the proof, if *Y *is a set of partial state paths, we use *P*(*Y*, *o'*|*O*^*i*^^-1^) in place of $\underset{\left(q_{i},\ldots ,q_{i+n-1}\right)\in Y}{\sum }P\left(q_{i},\ldots ,q_{i+n-1},o^{\prime }|O^{i-1}\right)$ for clarity.

(4) $$\begin{array}{c} \frac{\underset{\left(q_{i,\ldots ,}q_{i+n-1}\right)\in S^{n}}{\sum }P\left(q_{i},\cdots \;,q_{i+n-1},o^{\prime }|O^{i-1}\right)}{\underset{s\in S}{\sum }P\left(q_{i}=\cdots =q_{i+n-1}=s,o^{\prime }|O^{i-1}\right)}\\ =\frac{P\left(S^{n},o^{\prime }|O^{i-1}\right)}{\underset{s\in S}{\sum }P\left(S^{\left[s\right]},o^{\prime }|O^{i-1}\right)}\\ <\frac{P\left(S^{n},o^{\prime }|O^{i-1}\right)}{P\left(S^{\left[s_{x}\right]},o^{\prime }|O^{i-1}\right)}\\ =\overset{N}{\underset{j=1}{⋃}}\frac{P\left(S^{s_{j}},o^{\prime }|O^{i-1}\right)}{P\left(S^{\left[s_{x}\right]},o^{\prime }|O^{i-1}\right)}. \end{array}$$

Now we derive an upper bound of the contribution from state paths in $S^{s_{x}}$. In the following equations we make use of the fact that a state path with *k *non-self-transitions goes through at least $\frac{k}{2}$ non-s_*x *_states.

(5) $$\begin{array}{c} \frac{P\left(S^{s_{x}},o^{\prime }|O^{i-1}\right)}{P\left(S^{\left[s_{x}\right]},o^{\prime }|O^{i-1}\right)}\\ =\frac{\overset{n-1}{\underset{k=0}{\sum }}\underset{\tilde{S}\in S_{k}^{s_{x}}}{\sum }P\left(\tilde{S},o^{\prime }|O^{i-1}\right)}{P\left(S^{\left[s_{x}\right]},o^{\prime }|O^{i-1}\right)}\\ =\overset{n-1}{\underset{k=0}{\sum }}\underset{\tilde{S}\in S_{k}^{s_{x}}}{\sum }\frac{P\left(\tilde{S},o^{\prime }|O^{i-1}\right)}{P\left(S^{\left[s_{x}\right]},o^{\prime }|O^{i-1}\right)}\\ =\overset{n-1}{\underset{k=0}{\sum }}\underset{\begin{array}{c} \tilde{S}\in S_{k}^{s_{x}}\\ \tilde{S}=q_{i},\ldots ,q_{i+n-1} \end{array}}{\sum }\frac{P\left(q_{i}|O^{i-1}\right)e^{-{\left(\frac{o_{i}-\mu_{q_{i}}}{\sqrt{2\sigma }}\right)}^{2}}}{P\left(S_{x}|O^{i-1}\right)e^{-{\left(\frac{o_{i}-\mu_{s_{x}}}{\sqrt{2\sigma }}\right)}^{2}}}\\ \;\;\;\;\overset{i+n-2}{\underset{j=i}{\prod }}\frac{a_{q_{j}q_{j+1}}e^{-{\left(\frac{o_{j+1}-\mu_{q_{j+1}}}{\sqrt{2\sigma }}\right)}^{2}}}{a_{s_{x}s_{x}}e^{-{\left(\frac{o_{j+1}-\mu_{s_{x}}}{\sqrt{2\sigma }}\right)}^{2}}}\\ =\overset{n-1}{\underset{k=0}{\sum }}\underset{\begin{array}{c} \tilde{S}\in S_{k}^{s_{x}}\\ \tilde{S}=q_{i},\ldots ,q_{i+n-1} \end{array}}{\sum }\frac{P\left(q_{i}|O^{i-1}\right)}{P\left(S_{x}|O^{i-1}\right)}\overset{i+n-2}{\underset{j=1}{\prod }}\frac{a_{q_{j}q_{j+1}}}{a_{s_{x}s_{x}}}\\ \;\;\;\;\;\;\;\;\;\;\overset{i+n-1}{\underset{j=i}{\prod }}\frac{e^{-{\left(\frac{o_{j}-\mu_{q_{j}}}{\sqrt{2\sigma }}\right)}^{2}}}{e^{-{\left(\frac{o_{j}-\mu_{s_{x}}}{\sqrt{2\sigma }}\right)}^{2}}}\\ \leq \overset{n-1}{\underset{k=0}{\sum }}\underset{\begin{array}{c} \tilde{S}\in S_{k}^{s_{x}}\\ \tilde{S}=q_{i},\ldots ,q_{i+n-1} \end{array}}{\sum }\alpha {\left(\frac{1-t}{\left(N-1\right)t}\right)}^{k}\\ \;\;\;\;\;\overset{i+n-1}{\underset{j=i}{\prod }}\frac{e^{-{\left(\frac{o_{j}-\mu_{q_{j}}}{\sqrt{2\sigma }}\right)}^{2}}}{e^{-{\left(\frac{o_{j}-\mu_{s_{x}}}{\sqrt{2\sigma }}\right)}^{2}}}\\ \leq \overset{n-1}{\underset{k=0}{\sum }}\left(\begin{array}{c} n-1\\ k \end{array}\right){\left(N-1\right)}^{k}\alpha {\left(\frac{1-t}{{\left(N-1\right)}^{t}}\right)}^{k}{\left(e^{-\frac{d\tau }{\sigma^{2}}}\right)}^{\frac{k}{2}}\\ =\overset{n-1}{\underset{k=0}{\sum }}\left(\begin{array}{c} n-1\\ k \end{array}\right)\alpha {\left(\frac{1-t}{t}\right)}^{k}{\left(e^{-\frac{d\tau }{\sigma^{2}}}\right)}^{\frac{k}{2}}\\ =\overset{n-1}{\underset{k=0}{\sum }}\alpha \left(\begin{array}{c} n-1\\ k \end{array}\right){\left(\frac{1-t}{t}\right)}^{k}{\left(e^{-\frac{d\tau }{\sigma^{2}}}\right)}^{\frac{k}{2}}\\ =\overset{n-1}{\underset{k=0}{\sum }}\alpha \left(\begin{array}{c} n-1\\ k \end{array}\right){\left(rc\right)}^{k}\\ =\alpha {\left(1+rc\right)}^{n-1}. \end{array}$$

Similarly, we derive an upper bound of the contribution from state paths in $S^{s_{y}}$, where 1 ≤ *y *≠ *x *≤ *N*. Now we use the fact that, because of the pigeonhole principle any state path in $S^{s_{y}}$ has to go through at least $\frac{n}{N}$ non-s_*x *_states.

(6) $$\begin{array}{c} \frac{P\left(S^{s_{y}},o^{\prime }|O^{i-1}\right)}{P\left(S^{\left[s_{x}\right]},o^{\prime }|O^{i-1}\right)}\\ \leq \overset{n-1}{\underset{k=0}{\sum }}\underset{\begin{array}{c} \tilde{S}\in S_{k}^{s_{y}}\\ \tilde{S}=q_{i},\ldots ,q_{i+n-1} \end{array}}{\sum }\\ \alpha \left(\frac{1-t}{\left(N-1\right)t}\right)\overset{ki+n-1}{\underset{j=i}{\prod }}\frac{e^{-\frac{1}{2}{\left(\frac{o_{j}-\mu_{q_{j}}}{\sigma }\right)}^{2}}}{e^{-\frac{1}{2}{\left(\frac{o_{j}-\mu_{s_{x}}}{\sigma }\right)}^{2}}}\\ \leq \overset{n-1}{\underset{k=0}{\sum }}\left(\begin{array}{c} n-1\\ k \end{array}\right){\left(N-1\right)}^{k}\alpha {\left(\frac{1-t}{\left(N-1\right)t}\right)}^{k}{\left(e^{-\frac{d\tau }{\sigma^{2}}}\right)}^{\frac{n}{N}}\\ =\overset{n-1}{\underset{k=0}{\sum }}\left(\begin{array}{c} n-1\\ k \end{array}\right){\left(N-1\right)}^{k}\alpha {\left(\frac{1-t}{\left(N-1\right)t}\right)}^{k}{\left(e^{-\frac{d\tau }{\sigma^{2}}}\right)}^{\frac{n}{N}}\\ =\overset{n-1}{\underset{k=0}{\sum }}\left(\begin{array}{c} n-1\\ k \end{array}\right)\alpha {\left(\frac{1-t}{t}\right)}^{k}{\left(e^{-\frac{d\tau }{\sigma^{2}}}\right)}^{\frac{n}{N}}\\ =\overset{n-1}{\underset{k=0}{\sum }}\alpha \left(\begin{array}{c} n-1\\ k \end{array}\right){\left(\frac{1-t}{t}\right)}^{k}{\left(e^{-\frac{d\tau }{\sigma^{2}}}\right)}^{\frac{n}{N}}\\ =\overset{n-1}{\underset{k=0}{\sum }}\alpha c^{\frac{2n}{N}}\left(\begin{array}{c} n-1\\ k \end{array}\right)r^{k}\\ =\alpha c^{\frac{2n}{N}}{\left(1+r\right)}^{n-r}. \end{array}$$

Applying (5) and (6) in (4) we get,

$$\begin{array}{c} \frac{\underset{\left(q_{i},\ldots ,q_{i+n-1}\right)\in S^{n}}{\sum }P\left(q_{i},\ldots ,q_{i+n-1},o^{\prime }|O^{i-1}\right)}{\underset{s\in S}{\sum }P\left(q_{i}=q_{i+1}=\ldots =q_{i+n-1}=s,o^{\prime }|O^{i-1}\right)}\\ \leq \alpha \left({\left(1+rc\right)}^{n-1}+\left(N-1\right)c^{\frac{2n}{N}}{\left(1+r\right)}^{n-1}\right). \end{array}$$

**Note**: For simplicity of the notation, we follow the convention that $\mu_{x_{0}}=-\infty$ and $\mu_{x_{N+1}}=\infty$ so that the proof holds for *x *= 1 or *x *= *N*.
